# Magneto-transport Spectroscopy of the First and Second Two-dimensional Subbands in Al_0.25_Ga_0.75_N/GaN Quantum Point Contacts

**DOI:** 10.1038/srep42974

**Published:** 2017-02-22

**Authors:** Fangchao Lu, Ning Tang, Liangliang Shang, Hongming Guan, Fujun Xu, Weikun Ge, Bo Shen

**Affiliations:** 1 Department of Mathematics and Physics, North China Electric Power University, Beijing 102206, China; 2 State Key Laboratory of Artificial Microstructure and Mesoscopic Physics, School of Physics, Peking University, Beijing 100871, China; 3 Collaboration Innovation Center of Quantum Matter, Beijing 100871, China

## Abstract

Magnetic transport spectroscopy is investigated in quantum point contacts (QPCs) fabricated in Al_0.25_Ga_0.75_N/GaN heterostructures. The magnetic field perpendicular to the two-dimensional electron gas (2DEG) is shown to depopulate the quasi-one-dimensional energy levels in the first two-dimensional (2D) subband faster than those in the second one. In GaN based heterostructures, the energy levels in the second 2D subband is generally concealed in the fast course of depletion and hence rarely detected. The perpendicular magnetic field facilitates the observation of the second 2D subband, and provides a method to study the properties of these energy levels. A careful analysis on the rate of the magnetic depletion with respect to the level index and confinement is carried out, from which the profile of the lateral confinement in GaN based QPCs is found to be triangular. The stability diagram at 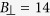
T shows the energy separation between the first and second 2D subband to be in the range of 32 to 42 meV.

III-Nitrides, especially GaN based heterostructures^1^, are attracting much interest in the fabrication of blue light emitting diode (LED) and high temperature, high frequency, and high power electronic devices. Furthermore, due to its relatively high mobility^2^,^3^, long spin lifetime^4^, and strong spin-orbit coupling (SOC)^5^,^6^ induced by the strong build-in electric field due to the large spontaneous and piezoelectric polarization^7^,^8^, the studies of spin related physics are also attractive. Quantum point contact (QPC) is a typical type of 1D system that can be realized based on two-dimensional electron gas (2DEG) in semiconductor heterostructures. Recent experiments proved an exciting possibility of using QPCs for spin injection and detection for spin field effect transistors^9^,^10^, yet little has been studied about the transport properties in GaN based QPCs. In this letter, the magneto-transport spectroscopy of the energy levels in the QPCs based on Al_0.25_Ga_0.75_N/GaN heterostructures was studied in magnetic fields up to 14 T. In a QPC with two two-dimensional (2D) subbands in the 2DEG occupied, the perpendicular field is found to depopulate the energy levels in the first 2D subband faster than those in the second one. At zero magnetic fields, the one-dimensional (1D) energy levels in the second 2D subband are generally concealed in the depletion process of the gated area. However, the application of high magnetic field is found to enable the observation of these energy levels, thus providing a method to study their properties.

## Methods

The Al_0.25_Ga_0.75_N/GaN heterostructures were grown on c-plane sapphire substrates by means of metal organic chemical vapor deposition (MOCVD). The electron density and mobility on the wafer reported in this letter are extracted from a Hall-bar, the values are 1.0 × 10^13^ cm^−2^ and 1.1 × 10^4^ cm^2^/Vs at 1.3 K respectively. The Al composition of the barrier layer used in the wafer, x = 0.25, was chosen to obtain a large polarization field, and thus a large Rashba spin-orbit coupling in the 2DEG, while preserving a relatively high electron mobility. Such structure forms a deep and narrow triangular quantum well at the hetero-interface, inducing a 2DEG with high carrier concentration, in which the electrons may even occupy the second subband^11^,^12^. Consequently, it takes a gate voltage of more than −8 V to deplete the 2DEG beneath the gate area, the influence of which appear not only in the measurements but also in the fabrication process.

**Figure 1:**
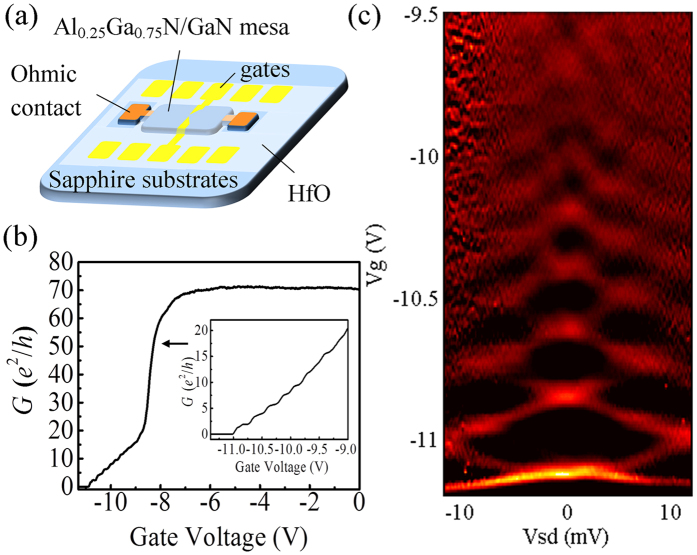
(**a**) Illustration of a QPC fabricated on Al_0.25_Ga_0.75_N/GaN heterostructures. (**b**) Linear-response conductance *G* of the QPC device measured against the gate voltage *V*_*g*_ at 1.3 K. The drop of the conductance indicated by the arrow represents the depletion of the 2DEG. The inset is the zoomed-in measurement of the quantum conductance, which shows up after the formation of the 1D channel below *V*_*g*_ = −9V. The series resistance, the current and bias offset from the preamplifier that was used in the measurements have all been subtracted from the original data. (**c**) Numerical derivative of the differential conductance of the QPC over the gate voltage, *d*^2^*I/dV*_sd_*dV*_g_, measured as functions of both *V*_*sd*_ and *V*_*g*_. The dark regions represent plateaus in the differential conductance, while the brighter regions represent the risings of the conductance in the transition regions between plateaus.

For the details of the growth and fabrication process, please refer to ref. 13. Figure 1(a) shows an illustration of a typical QPC structure designed for this measurement. Ohmic contact of the heterostructure is realized by the Ti/Al/Ni/Au alloy, and the dielectric layer is 20 nm of amorphous HfO_2_. It is worth to mention that the device measured in this letter is fabricated with wedge-shaped gates, in which the angle of the tips falls in the range of 30° and 45°, and the separation between the gates is around 50 nm. A series of split-gates with other shapes have been tested in our measurements, however, in the case of GaN based heterostructures, i.e. with 2DEGs difficult to deplete, asymmetric wedge-gates are proved to be the best design to obtain stable quantized conductance. A brief summary of the measured QPCs is given in the supplemental material.

## Results and Discussion

The measurements were performed at 1.3 K with a *dc* measurement setup. As the gate voltage is increased negatively, the 2DEG beneath the gates is depleted, and a drastic drop in the current marks the formation of the 1D channel, as shown in Fig. 1(b). Quantized conductance plateaus can be observed subsequently in the linear transport regime. The inset of Fig. 1(b) shows the conductance of the QPC with a source-drain bias of *V*_sd_ = 100 μV. Differential conductance (*G*) in the nonlinear regime is obtained with respect to the source-drain voltage (*V*_sd_) and gate voltage (*V*_g_). In order to highlight the change in *G*, d*G*/d*V*_g_ are derived and plotted in Fig. 1(c). The yellow/red curves in the figure mark the status that the chemical potential of the source or drain is aligned with a certain energy level in the QPC. Lever arm factors of the split-gates can be extracted for the first few 1D energy levels from the figure; and from the slopes of the curves, one can also inspect that for a particular energy level, the lever arm decreases as the gate voltage is swept towards zero.

**Figure 2:**
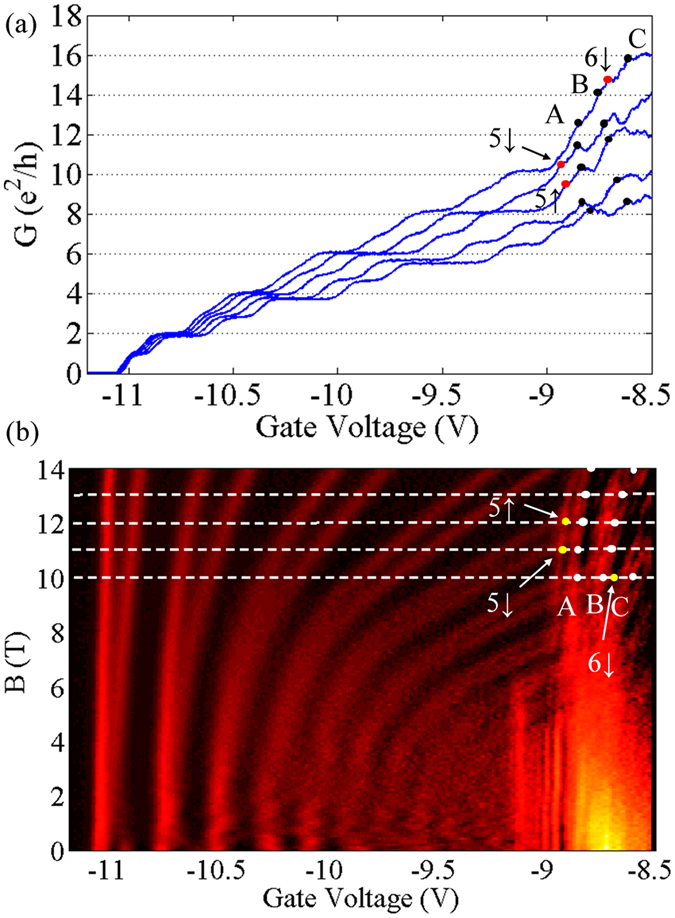
(**a**) The conductance of the QPC as a function of the gate voltage. (**b**) Numerical derivative of the conductance *G* of the QPC over the gate voltage *V*_g_, plotted as the function of perpendicular magnetic field *B* and *V*_g_. From top to bottom, the curves in (**a**) correspond to *B* = 10, 11, 12, 13, 14 T respectively, as high-lighted in (**b**) by white-dashed lines. The black dots in (**a**) correspond to the white dots in (**b**); and the red to the yellow.

The magneto-transport properties of the QPC are investigated by measuring the conductance of the QPC under various magnetic fields. Figure 2(b) shows the derivative of the conductance (*G*) with respect to *V*_g_, measured with *V*_sd_ = 100 μV, in the magnetic fields *B* perpendicular to the 2DEG. In the 2D colour plot, the bright lines represent the alignment of the Fermi level with an individual 1D energy level, and the splitting of the lines with increasing *B* is a manifest of the Zeeman Effect in a certain orbital level. Besides the Zeeman splitting that can occur in any field orientation, a bending of the bright lines toward smaller negative gate voltage is found, which is related solely to the perpendicular component of the magnetic field. In the 2DEG based QPCs, the effect of the perpendicular magnetic field is equivalent to an additional parabolic lateral confinement. The electrostatic confinement in QPCs is usually assumed to be harmonic, i.e. 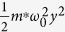, thus the additional magnetic confinement increases the energy spacing from 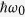 to 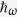, in which 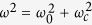, and 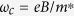 is the cyclotron frequency. Consequently, the increasing magnetic field serves to depopulate the higher energy levels^14^, leading to the shifting of the bright lines. In Fig. 2(b), two groups of bright lines emerge: one in the low energy regime in the left part of the figure, with slopes getting smaller with increasing *B* and energy level index; and the other group extended out from the bright region down to the right of the figure, with larger slopes than the first-group lines crossing with them. Clearly, the first group of lines represent the energy levels in the first 2D subband, and similar signals have been reported in other 2DEG based QPCs^15^. These energy levels are marked in the figure by *n*↑(↓), in which *n* stands for the index of the 1D energy level and ↑(↓) for the state of spin. The second group of lines are marked with A, B and C. These lines cannot be discerned in zero magnetic fields, since they are obscured by the fast rising of the conductance indicated by the arrow in Fig. 1(b). However, they are found to shift less with increasing *B*, and become consequently observable in high fields.

To analyze the nature of these lines, the conductance at *B* = 10, 11, 12, 13, 14 T, indicated in Fig. 2(b) by white dashed lines, are plotted in Fig. 2(a). It should be noted that, the curves from top to bottom correspond to increasing *B* from 10 to 14 T respectively, due to the effect of the magnetic depopulation. Each white dot in Fig. 2(b) corresponds to a black dot in Fig. 2(a), marking the left edge of a conductance plateau when A, B or C crosses with the white dashed lines. The left part of the curves in Fig. 2(a) show regular conductance plateaus of *e*^2^/*h*, contributed by the first-group levels in the low energy regime; while in the high energy regime, the conductance plateaus become irregular, due to the appearance of A, B and C and their crossing with the first-group levels. Despite of this, for each magnetic field, the steps in conductance induced by A, B and C can be read out from Fig. 2(a) through a careful analysis, the detail of which is included in the supplemental material. It can be concluded from the analysis that the bright lines A, B and C all correspond to the change in *G* of *e*^2^/*h*, confirming their nature as signals of spin-split 1D energy levels. It should be noted that, in Fig. 2(a), the steps of conductance are not exactly *e*^2^/*h* for any of the energy levels in the high energy regime. This is because when there are a large number of energy levels occupied, transport through these levels is complicated by scattering. For example, the conductance of the system could be suppressed by back scattering^16^. Also, when the energy difference between two adjacent levels is small, the higher level may also contribute in the conductance via excitation, inducing a diversion from the exact quantized conductance *e*^2^/*h*.

It is reasonable to deduce that the second group of bright lines are from the second subband in the 2DEG. In the following section, we note the energy levels by (*m, n, σ*), in which *σ* = ↑(↓) represent the spin of the energy level, and *m, n* represent the quantum numbers in the perpendicular and lateral confinements respectively. It is worth to mention that, first, a certain *V*_g_ induces the same lateral electrostatic confinement to the electrons in both the first and second 2D subbands, therefore the lever arm *α* for the *n*th 1D energy level in both 2D subbands should be the same. Thus, we can express the lever arm factors by 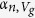. Second, electrons in both 2D subbands in Al_x_Ga_1-x_N/GaN heterostructures have almost the same effective mass, as is proved by the magneto-intersubband oscillations of the 2DEG^11^. For an (*m, n*) subband in 2DEGs, we derive the slope at a certain *B* and *V*_g_ as:


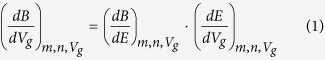


The last term on the right of the equation represents the lever arm factor of the energy level (*m, n*) at *V*_g_, i.e. 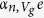. Neglecting the Zeeman splitting for simplicity, and assuming the lateral electrostatic confinement to be parabolic, the magnetic field enhances the energy of the *n*th level from 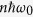 to 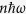. Therefore we obtain


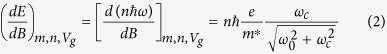


Combining equations (1) and (2) gives


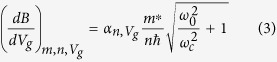


The above equation explains the evolution of the bright lines, i.e. the slope gets smaller for higher *n* and/or in higher *B*. Note that adding the Zeeman term into the equation does not affect this conclusion.

In Fig. 2(b), each curve stemming from a certain energy level can be inspected in three regimes. When *B* is large enough, the total confinement is dominated by the magnetic confinement, i.e. 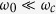, and Eq. (3) can be written as 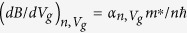. This corresponds to the upper right region of the curves, in which the slopes do not vary much with *V*_*g*_. On the other hand, when *B* is small and the electric confinement is dominating, i.e. 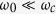, and Eq. (3) can be written as 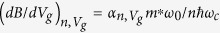. This corresponds to the steep part of the curves, in which the slopes are approximately inversely proportional to *B*, tending to infinity when B approaches zero. Along a particular curve, as B increases from 0 to 14 T, *ω*_c_ is increasing while *ω*_0_ decreasing. Between those two regimes, a prominent change of slope, which is most obvious in the curves of the (1, 3, ↑(↓)) to (1, 6, ↑(↓)) levels, marks the region when *ω*_0_ and *ω*_c_ are comparable. For energy levels with *m* = 1, as *n* gets larger, the magnetic field at which the “turning point” appears gets smaller, agreeing with the decreasing trend of the confinement *ω*_0_. One should note that the above equations are deduced under the assumption of a parabolic confinement, however, the potential defined by the split gates are not quite parabolic, and *ω*_0_ can only be viewed as an equivalent confinement parameter. This can be known from the qualitative comparison between the curves of (2, 1, ↑(↓)), and (1, 5, ↑(↓)). It is clear in Fig. 2(b) that the “turning point” of (2, 1, ↑(↓)) appears around or even above *B* = 14 T; while for (1, 5, ↑(↓)), which crosses with the former at around *B* = 12 T, the “turning point” appears between *B* = 6 to 8 T. Thus, at the crossing point around B = 12 T, we have 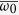
(2, 1, ↑(↓))
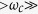

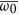
(1, 5, ↑(↓)). As mentioned in the previous discussion, in regard to the lateral confinement, we can presume 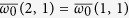, neglecting the influence of the Zeeman splitting. Then it is clear that at the crossing point of the two lines, (with *V*_*g*_ biased between −9 and −8.5 V,) we have 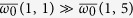. Similar inference can be made for 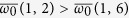. It is known that for an ideal harmonic confinement, *ω*_0_ should be the same for all the energy levels. The above result shows the fact that the level spacing for the lower levels are larger than those for higher levels, suggesting the confinement to be a triangular quantum well instead of a parabolic or square one, which has been commonly used in modeling.

To explore more of the information provided by the slopes of the curves, we inspect the two energy levels crossing at the same *B* and *V*_g_, e.g. curves (1, 5, ↑) and (2, 1, ↑) at their crossing point of *B* = 12.2 T and *V*_g_ = 8.92 V. In this case, the lever arms are usually different for the two levels, according to Eq. (3),


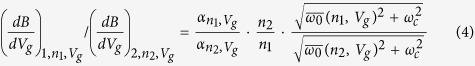


In this example, at *B* = 12.2 T and *V*_g_ = 8.92 V,


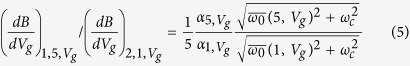


From Fig. 2(b), the slopes of both curves at the crossing point are extracted to be 4.6 and 67 T/V respectively; the latter is more than one magnitude larger than the former. From the previous discussion, it is known that 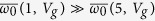, also, the lever arm for the first energy level should be larger than those for the higher levels. Therefore, it can be concluded that the experimental result in Fig. 2(b) agrees with our inference from Eq. (3).

We can also investigate the curves (1, 1, ↑), (1, 1, ↓), (2, 1, ↑), and (2, 1, ↓) at the same magnetic field *B* = 13.5 T. The slopes are approximately obtained by the line segments between the crossing points at *B* = 13 and 14 T, which are listed in Table 1. From the table, one can see that the slopes of (1, 1, ↑) and (1, 1, ↓) are approximately four times those of (2, 1, ↑), and (2, 1, ↓). We obtain from the above equation the ratio of the slopes for the same *n* in the first and second subbands as:


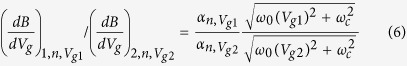


Here *V*_g1_ and *V*_g2_ represent the gate voltage for the (1, *n*) and (2, *n*) levels to cross the Fermi level. The above equations show that, at the same magnetic field, the ratio of the slopes is determined by the ratio of α at different gate voltages, at which either of the energy levels meets the Fermi level, and the ratio of the total confinement. For *n* = 1 and *B* = 13.5 T, it can be known from the previous discussion that for *n* = 1 in either the first 2D subband or the second, the electrostatic confinement *ω*_0_ is larger than the magnetic confinement *ω*_*c*_, thus, Eq. (4) can be written approximately as


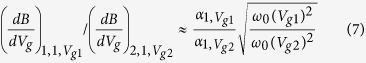


Thus, the ratio 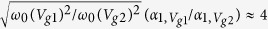 can be derived for the spin-up levels (1, 1, ↑) and (2, 1, ↑), as well as spin-down levels. As mentioned earlier in this letter, the lever arm α, as well as the electrostatic confinement *ω*_0_, decrease as the gate voltage is swept toward zero. This trend is consistent to the ratio obtained in the experiment, and one can deduce that the confinement parameter *ω*_0_(*V*_*g*1_) is no larger than four times of *ω*_0_(*V*_*g*2_). Since the lever arm factor α is related to *ω*_0_, the relations between *ω*_0_ and *V*_g_ can be obtained by numerical analysis. Furthermore, the profile of the lateral confinement can also be revealed through a simulation of the magnetic depopulation, based on our discussion.

The rate of magneto-depopulation for the 1D energy levels in the first and second 2D subbands is different. With the same total energy, the energy levels from the first 2D subband have larger level index than those from the second. For them, the magnetic depopulation with increasing *B* is much faster than the levels from the second subband, which have smaller level index. In Al_0.25_Ga_0.75_N/GaN heterostructure with narrow triangular quantum well at the interface, at *B* = 0 T, the electrons in the second subband usually get depleted before the channel becomes narrow enough for the observation of the quantized conductance. Consequently, with increasing perpendicular magnetic field, the transport signals for energy levels from the second 2D subband stem from the steep part in the curve of the conductance as shown in Fig. 1(b) and the bottom line of Fig. 2(b), and finally appear among the first few observable energy levels at *B* = 14 T. Salis *et al*. reported in GaAs based 1D electron waveguides^17^, that when an in-plane field perpendicular to the waveguide is applied, the second 2D subband is depleted faster than the first one with increasing magnetic field. It is clear in the comparison, that although both measurements are carried out in a field perpendicular to the momentum of electrons, the in-plane field (*B*_*y*_) and out-of-plane field (*B*_*z*_) depletes the 2D subbands in different ways. Namely, *B*_*y*_ increases the spacing between the first and second 2D subbands, while *B*_*z*_ enlarges the spacing between the 1D sub-levels in both 2D subbands.

**Figure 3:**
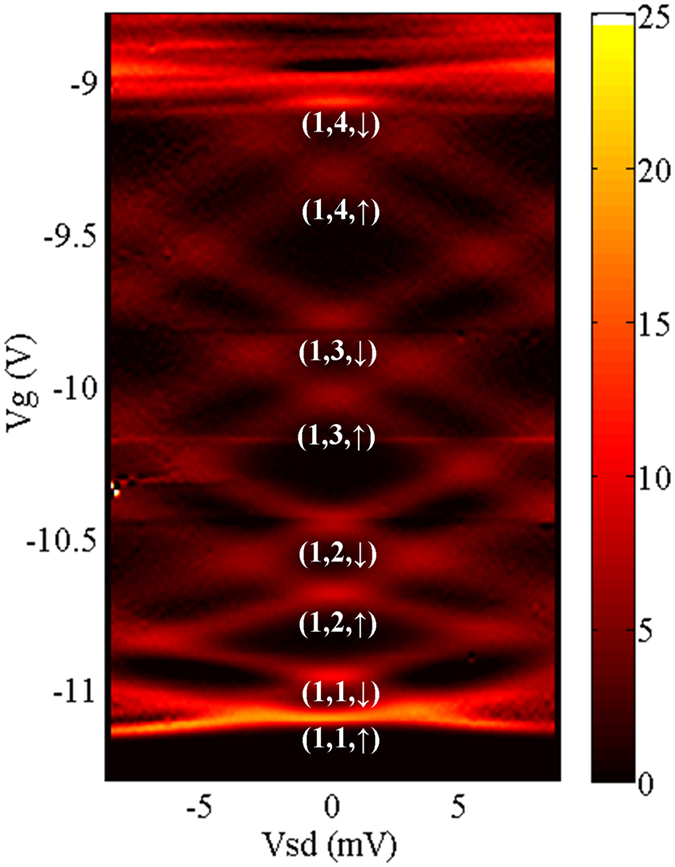
Numerical derivative of the differential conductance of the QPC over the gate voltage, *dI*^2^/*dV*_*sd*_*dV*_g_, measured in a perpendicular magnetic field *B* = 14 T, and plotted as functions of both *V*_*sd*_ and *V*_*g*_. The energy levels are marked below the corresponding curves.

It is interesting to compare the stability diagram of the QPC with and without the perpendicular magnetic fields, i.e. Figs 1(c) and 3. In Fig. 1(c), the energy spacings between adjacent levels are shown to decrease prominently from lower to higher levels, before the gate voltage of 9.5 V. And then in the region between 9.5 V to 9 V, the energy spacings are obscured and adjacent levels are almost merged together, which is shown in the bottom line of Fig. 2(b). This is due to the widening of the potential well, while the negative *V*_g_ is decreased. However, in Fig. 3, the orbital energy spacing does not vary as much from the first to the fourth levels. This shows that the decrease of the negative *V*_g_ does not affect the profile of the total confinement as much as in the previous case. In the diamond structure of the fourth orbital levels, the pattern just above the higher spin level, i.e. (1, 4, ↓), is disrupted by the appearance of the second 2D subband, which can also be seen in the upper line of Fig. 2(b) from 9 to 8.5 V. As discussed before, the total confinement is composed of the triangular quantum well defined by the split gates, and the magnetic confinement which can be viewed as a harmonic quantum well. At *B* = 14 T, the magnetic confinement dominates the total confinement for most of the energy levels, leading to the almost even orbital level spacings. The Zeeman splitting from the first to the fourth levels in the first 2D subband shows a decreasing trend, which agrees with our previous study^13^. The orbital level spacing is also decreasing. One can read from Fig. 3, that the energy spacing between (1, 1, ↑) and (1, 1, ↓) is around 4 meV, and that between (1, 1, ↓) and (1, 2, ↑) is in the range of 6 to 7 meV. Similarly, the energy spacings from (1, 1, ↑) to (1, 4, ↓) can all be obtained from Fig. 3. The detailed values are noted on Fig. S2 in the supplemental material, and the sum of which is around 32 meV. From Fig. 3, the lower limit of the energy spacing between the first and second 2D subbands to be 32 meV. Meanwhile, since the energy between (1, 4, ↓) and (1, 5, ↓) cannot be larger than that between (1, 3, ↓) and (1, 4, ↓), which is in the range of 9 to 10 meV, the upper limit of the energy spacing between (1, 1, ↑) and (1, 5, ↓) should be 42 meV. According to Fig. 2(b), at *B* = 14 T, the energy levels (2, 1, ↑) and (2, 1, ↓) fall in between (1, 4, ↓) and (1, 5, ↓). Therefore, the energy separation between the first and second 2D subband, i.e. (1, 1, ↑(↓)) and (2, 1, ↑(↓)), is in the range of 32 and 42 meV. It is worth pointing out that the energy spacing between the first and second 2D subband is not affected by the magnetic field, thus the above result can be extended to the case of zero field. The stability diagram in high magnetic field also shed some light on other properties of the second 2D subband, such as the effective *g* factors. Please refer to the supplemental material for the detailed information.

In conclusion, the magneto-transport spectroscopy of the subbands in Al_0.25_Ga_0.75_N/GaN based QPCs is investigated. The magnetic field perpendicular to the 2DEG plane depletes the 1D energy levels from the first subband faster than those from the second one, thus facilitates the observation and study of the electron properties in the second 2D subbands, which is otherwise inaccessible. The depopulation process shown in the spectroscopy agrees well with theoretical analysis, providing a method to analyze the profile of the lateral confinement and energy level distributions of GaN based QPCs numerically, which may be used in exploring the spin-orbit coupling and the application of GaN based QPCs in spin injections. The lateral electrostatic confinement is found to be triangular from the spectroscopy, instead of the parabolic or square shape that is commonly used in the modeling. Also, the level spacing between the first and second 2D subband can be obtained by analysis of the stability diagram in perpendicular magnetic fields, which is in the range of 32 to 42 meV.

## Additional Information

**How to cite this article:** Lu, F. *et al*. Magneto-transport Spectroscopy of the First and Second Two-dimensional Subbands in Al_0.25_Ga_0.75_N/GaN Quantum Point Contacts. *Sci. Rep.* **7**, 42974; doi: 10.1038/srep42974 (2017).

**Publisher's note:** Springer Nature remains neutral with regard to jurisdictional claims in published maps and institutional affiliations.

## Supplementary Material

Supplementary Information

## Figures and Tables

**Table 1:** The slopes of the energy levels at *B* = 13.5 T, extracted from Fig. 2(b).

Energy levels	*V*_g_ at *B* = 13 T(V)	*V*_g_ at *B* = 14 T(V)	d*V*_g_/d*B* (V/T)
(1,1,↑)	−11.020	−11.010	0.010
(1,1,↓)	−10.895	−10.880	0.015
(2,1,↑)	−8.885	−8.845	0.040
(2,1,↓)	−8.670	−8.625	0.060
