# Supplementary material for: Magneto-transport Spectroscopy of the First and Second Two-dimensional Subbands in Al0.25Ga0.75N/GaN Quantum Point Contacts
Source: Sci Rep. 2017 Feb 22;7:42974. doi: 10.1038/srep42974 (PMC5320474; doi:10.1038/srep42974)
Supplement: Supplementary Information [file srep42974-s1.doc]

Magnetic transport spectroscopy of the first and second two-dimensional sub-band in Al_0.25_Ga_0.75_N/GaN quantum point contacts

Fangchao Lu,^1,2^ Ning Tang,^2,3*^ Liangliang Shang, ^2^ Hongming Guan,^2^ Fujun Xu,^2^ Weikun Ge,^2^ Bo Shen2,3[[1]](#footnote-2)*

^1^Department of Mathematics and Physics, North China Electric Power University, Beijing 102206, China

^2^State Key Laboratory of Artificial Microstructure and Mesoscopic Physics, School of Physics, Peking University, Beijing 100871, China

^3^Collaboration Innovation Center of Quantum Matter, Beijing 100871, China

KEYWORDS: quantum point contacts (QPCs), Al_0.25_Ga_0.75_N/GaN heterostructures magnetic depopulation, the second two-dimensional subband

^*^ E-mail address: [ntang@pku.edu.cn](mailto:ntang@pku.edu.cn), [bshen@pku.edu.cn](mailto:bshen@pku.edu.cn),

**S1. A brief summary of designs of QPCs for Al_0.25_Ga_0.75_N/GaN heterostructures**

We have fabricated and measured QPCs with either needle-shaped gates or wedge-gates with different angles. Fig. S1 shows several examples of the typical QPCs. The needle-shaped split-gates, as in Fig. S1(a), can only pinch off the QPC at gate voltages larger than 20 V, such gate voltage causes instability in the HfO_2_ dielectric layer. The rate of depletion is correlated with the area of the gates, to reduce the gate voltage required for pinch-off, wedge-shaped design are more preferable for GaN based QPCs. On the other hand, the symmetrical wedge-gates, as shown in Fig. S1 (d), are also ruled out, because the contour of most gates tends to be irregular, possibly due to the proximity effect. The single-sided wedge-gates are more controllable in both fabrication and measurement. In such QPCs, split-gates with tips in the range of 30^o^ and 45^o^, and channel width within the range of 50 and 80 nm, produce the best rate of depletion in the Al_0.25_Ga_0.75_N/GaN heterostructures.


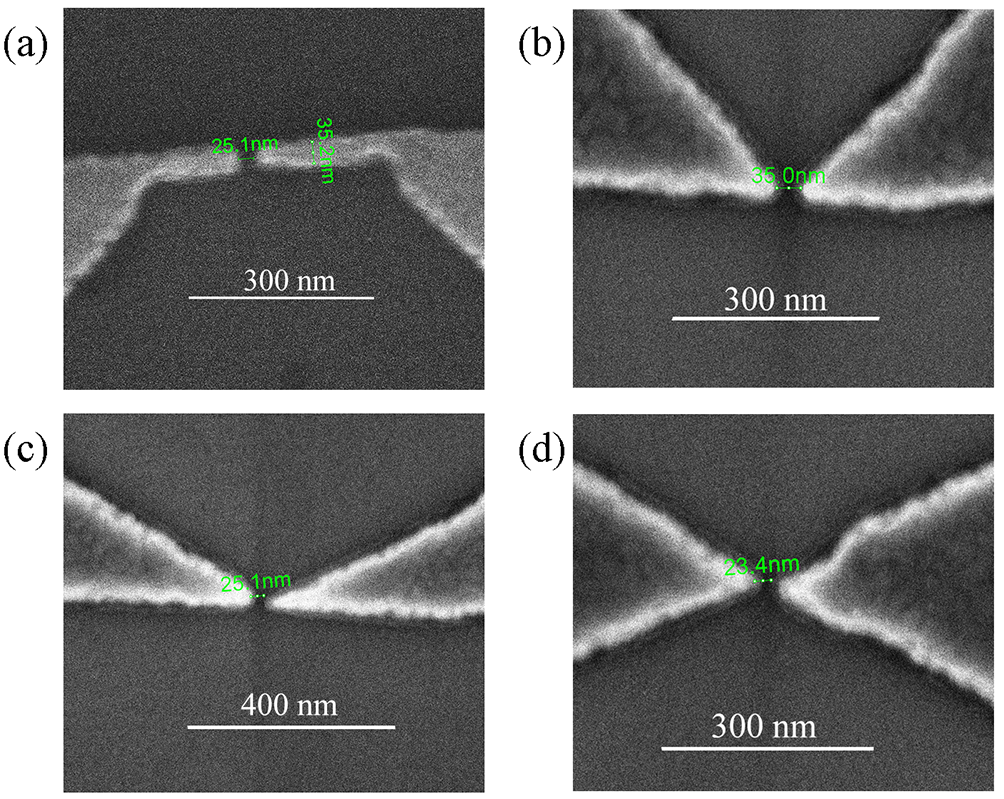


Figure S1. SEM images of several QPC devices tested in this work. (a) Needle-shaped split-gates. (b) Wedge-shaped split-gates with tips of about 45^o^. The actual shape is sharper than the designed pattern. (c) Wedge-shaped gates with tips of about 27^o^. (d) Symmetrical wedge-shaped gates with tips of about 55 ^o^.

**S2. Detailed analysis of Fig. 2 (a), concerning the quantum conductance of the QPC at magnetic fields B = 14, 13, 12, 11 and 10 T.**

1. At *B* = 14 T, as the conductance channel opens, the sequence of the bright lines is 4↓ → A → B → (5↑). The change of conductance is 0.9*e*^2^/*h* from 4↓ to A, and 0.4*e*^2^/*h* from A to B.
2. At *B* = 13 T, the sequence of the bright lines is 4↓ → A → the crossing of B and 5↑ → (5↓). The change of conductance is 0.8*e*^2^/*h* from 4↓ to A, and 1.4*e*^2^/*h* from A to the crossing of B and 5↑.
3. At *B* = 12 T, the sequence of the bright lines is 4↓ → 5↑ → A → the crossing of 5↓ and B → (C). The change of conductance is 1.1*e*^2^/*h* from 4↓ to 5↑, 1.0*e*^2^/*h* from 5↑ to A, and 1.6*e*^2^/*h* from A to the crossing of 5↓ and B.
4. At *B* = 11 T, the sequence of the bright lines is 4↓→ 5↓ → A → the crossing between B and 6↑ → (C). The change of conductance is 1.5*e*^2^/*h* from 4↓ to 5↓, 0.9*e*^2^/*h* from 5↓ to A. From A to the crossing of B and 6↑, the change of conductance is 1.3*e*^2^/*h* (black point) or 1.6*e*^2^/*h* (red point).
5. At B = 10 T, the sequence of the bright lines is 5↓ → the crossing between A and 6↑ → B → 6↓→ C. Since there are multiple crossings in this region, the borders between adjacent conductance plateaus are not so clear. However, we can still find in Fig. 2 (a) the corresponding position of the bright lines in Fig. 2 (b), and observe the minor change in slope at those positions. The change of conductance is 2.1*e*^2^/*h* from 5↓ to the crossing of A and 6↑, 1.9*e*^2^/*h* from A to B, 0.9*e*^2^/*h* from B to 6↓, and 1.0*e*^2^/*h* from 6↓ to C.

**S3. Obtaining information about the first and second 2D subbands from Fig. 3**

The stability diagram at $B_{\perp}=14$T provides us a potential method to explore the properties of the second 2D subband of Al_0.25_Ga_0.75_N/GaN heterostructures. The energy separations between adjacent levels can be read out from the diagram in Fig.3. Here, we replot the diagram in Fig. S2. From bottom to top, the energy levels are marked by (1, 1, ↑), (1, 1, ↓), …, (1, 4, ↓), and the energy level separations between adjacent levels are noted on the figure. The energy separation between (1, 1, ↑) and (1, 4, ↓) is thus obtained to be 32 V. The energy between (1, 4, ↓) and (1, 5, ↓) cannot be larger than that between (1, 3, ↓) and (1, 4, ↓), which is in the range of 9 to 10 meV, thus, the upper limit of the energy spacing between (1, 1, ↑) and (1, 5, ↓) is 42 meV. According to Fig. 2 (b), at B = 14 T, the energy levels (2, 1, ↑) and (2, 1, ↓) fall in between (1, 4, ↓) and (1, 5, ↓). Therefore, the energy separation between the first and second 2D subband, i.e. (1, 1, ↑(↓)) and (2, 1, ↑(↓)), is in the range of 32 and 42 meV.

In Fig. 3 of the main text, the transport signal between *V*_g_ = -9 V and -8.5 V is not quite regular, due to the appearance of the second 2D subband. One can also see this from Fig. 2, in which the energy levels (2, 1, ↑) and (2, 1, ↓) are broadened, and (2, 1, ↑) is quite close to the energy level (1, 4, ↓), consequently, the patterns around and above (1, 4, ↓) is just vaguely discernable. In Fig. S2, we added white dashed lines to the vague diamonds of (2, 1, ↑) and (2, 1, ↓), as a guide to the eyes. From these diamond patterns, one can read that the Zeeman splitting energy between (2, 1, ↑) and (2, 1, ↓) is no less than 5 meV. Because of the broadening of the energy levels and the noise, it is difficult to obtain the exact value of the effective *g* factors in the second 2D subband from these data. However, this work suggests that the effective *g* factor in the second 2D subband is no smaller, even larger, than that in the first subband.


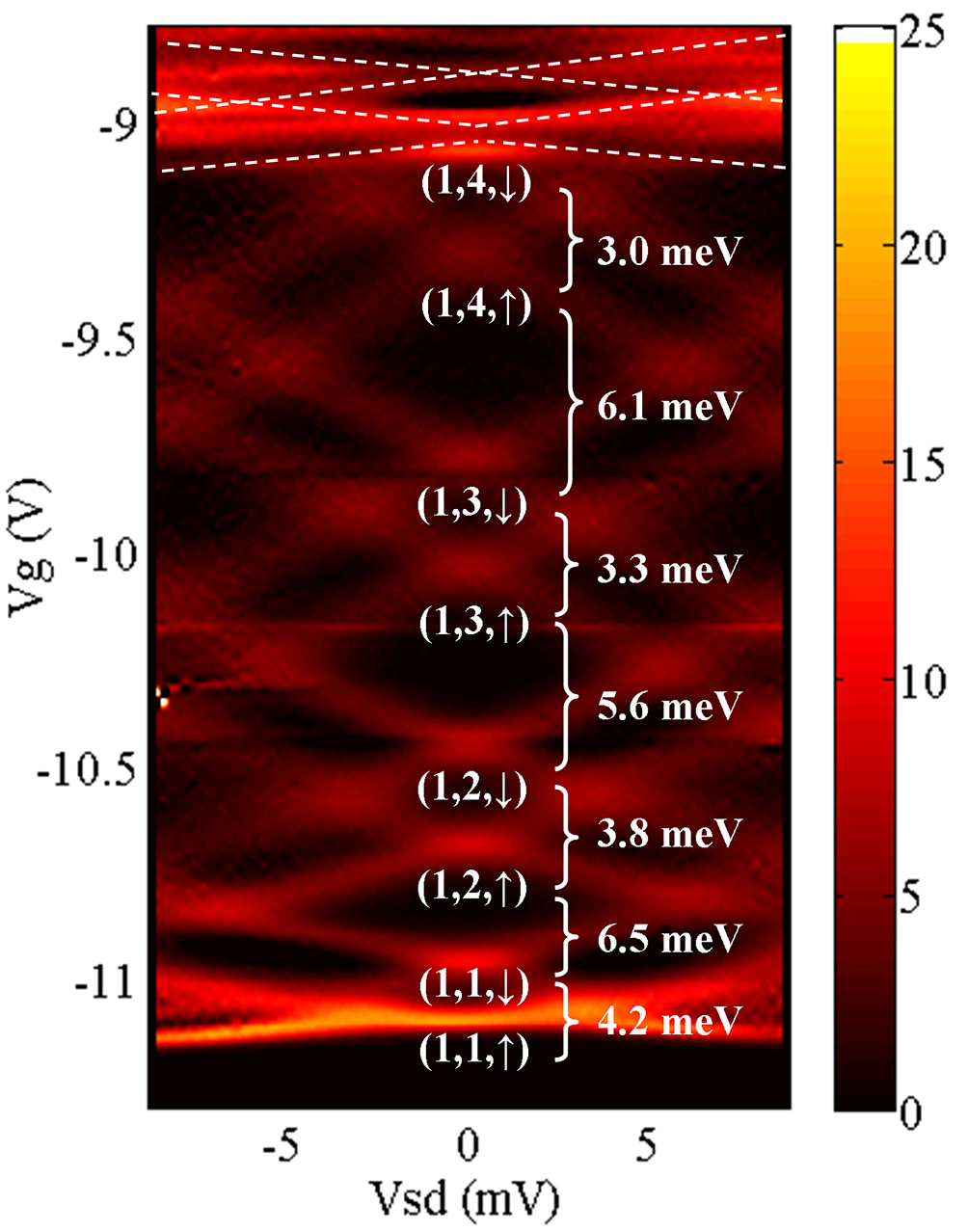


Figure S2. Numerical derivative of the differential conductance of the QPC over the gate voltage,$d^{2}I/dV_{sd}dV_{g}$, measured in a perpendicular magnetic field *B* = 14 T, and plotted as functions of both *V*_sd_ and *V*_g_. The crossing points around *V*_sd_ = 0 V corresponds to the situations when an (m, n, ↑(↓)) level is aligned with both source and drain. From (1, 1, ↑) to (1, 4, ↓), the energy level separations between adjacent levels are noted on the figure. And white dashed lines are added to the diamonds of (2, 1, ↑) and (2, 1, ↓) as a guide to the eyes.

1. ^^ [↑](#footnote-ref-2)
